# Effects of Step-Wise Increases in Dietary Carbohydrate on Circulating Saturated Fatty Acids and Palmitoleic Acid in Adults with Metabolic Syndrome

**DOI:** 10.1371/journal.pone.0113605

**Published:** 2014-11-21

**Authors:** Brittanie M. Volk, Laura J. Kunces, Daniel J. Freidenreich, Brian R. Kupchak, Catherine Saenz, Juan C. Artistizabal, Maria Luz Fernandez, Richard S. Bruno, Carl M. Maresh, William J. Kraemer, Stephen D. Phinney, Jeff S. Volek

**Affiliations:** 1 Department of Kinesiology, University of Connecticut, Storrs, CT, United States of America; 2 Nutritional Sciences Department, University of Connecticut, Storrs, CT, United States of America; 3 Department of Human Sciences, Ohio State University, Columbus, OH, United States of America; 4 School of Medicine (emeritus), University of California Davis, Davis, CA, United States of America; University of Nebraska Medical Center, United States of America

## Abstract

Recent meta-analyses have found no association between heart disease and dietary saturated fat; however, higher proportions of plasma saturated fatty acids (SFA) predict greater risk for developing type-2 diabetes and heart disease. These observations suggest a disconnect between dietary saturated fat and plasma SFA, but few controlled feeding studies have specifically examined how varying saturated fat intake across a broad range affects circulating SFA levels. Sixteen adults with metabolic syndrome (age 44.9±9.9 yr, BMI 37.9±6.3 kg/m^2^) were fed six 3-wk diets that progressively increased carbohydrate (from 47 to 346 g/day) with concomitant decreases in total and saturated fat. Despite a distinct increase in saturated fat intake from baseline to the low-carbohydrate diet (46 to 84 g/day), and then a gradual decrease in saturated fat to 32 g/day at the highest carbohydrate phase, there were no significant changes in the proportion of total SFA in any plasma lipid fractions. Whereas plasma saturated fat remained relatively stable, the proportion of palmitoleic acid in plasma triglyceride and cholesteryl ester was significantly and uniformly reduced as carbohydrate intake decreased, and then gradually increased as dietary carbohydrate was re-introduced. The results show that dietary and plasma saturated fat are not related, and that increasing dietary carbohydrate across a range of intakes promotes incremental increases in plasma palmitoleic acid, a biomarker consistently associated with adverse health outcomes.

## Introduction

Current dietary guidelines recommend that Americans decrease saturated fat to 7–10% of total energy and to consume the majority of calories from carbohydrate [1]. To accomplish these goals requires limiting whole foods that contain saturated fat (e.g., beef, eggs, high-fat dairy). A reduction in dietary saturated fat typically results in greater carbohydrate intake. The metabolic and clinical effects of replacing dietary saturated fat with carbohydrate are complicated and vary from person-to-person, but on a population level such a dietary manipulation may have untoward outcomes [2].

A consequence of consuming dietary sugars and starches above levels that can be directly oxidized is that a greater proportion is converted to fat (i.e., *de novo* lipogenesis). De novo lipogenesis (DNL) increases several-fold when carbohydrate is fed above energy needs [3], but isocaloric high-carbohydrate diets [4] and high-carbohydrate meals [5], [6] also promote DNL and hypertriglyceridemia in individuals with insulin resistance. The major product of DNL is palmitate (16∶0), a saturated fatty acid (SFA), but monounsaturated fatty acids (MUFA) are also formed as a result of desaturation, most notably palmitoleic acid (cis-16:1n-7). Healthy men overfed carbohydrate showed increased very low-density lipoprotein-triglyceride (VLDL-TG) palmitic (28.3 to 37.9%) and palmitoleic (3.8 to 10.0%) acids [3]. Thus, high-carbohydrate intake in individuals with an impaired ability to oxidize glucose stimulates DNL and secretion of SFA- and MUFA-enriched VLDL particles; a serum profile associated with insulin resistance [7].

In observational studies, the most consistent nonessential fatty acid that predicts metabolic syndrome [8], [9] and type-2 diabetes [10]–[14] is palmitoleic acid measured in erythrocytes, plasma cholesteryl ester (CE), or plasma phospholipid (PL). Palmitic and total SFA are usually significant predictors as well. In non-diabetic men that were followed for 5 yr, higher proportions of erythrocyte palmitoleic acid were significantly associated with worsening of hyperglycemia [15]. Higher proportions of palmitic acid and total SFA in blood lipids are associated with increased risk for heart disease [16]–[20] and more recently cancer [21]. Thus, a large body of evidence indicates that higher proportions of blood SFA and palmitoleic acid are associated with the pathophysiology of glucose intolerance and cardiovascular disease.

It is commonly believed that circulating fatty acids reflect dietary intake, but the associations are weak, especially for SFA and MUFA. In controlled isocaloric or hypocaloric experiments, when dietary carbohydrate is reduced, circulating levels of lipogenic fatty acids (i.e., palmitoleic, palmitic and total SFA) consistently decrease, despite higher saturated fat intake [22]–[25]. Proportionately, palmitoleic acid is the most responsive fatty acid to carbohydrate overfeeding [3] and it drops precipitously when carbohydrate is limited to less than 50 g/day [24], [25]. The results of these studies provide credible evidence that plasma SFA correlates poorly with dietary saturated fat and better with carbohydrate, and that plasma palmitoleic acid in particular is metabolically aligned with processing of dietary carbohydrate.

Although accumulation of SFA in circulating lipid fractions appears to be modulated by carbohydrate more than dietary saturated fat, there are no controlled studies examining this premise across multiple levels of carbohydrate in the same person. The aim of this study was to determine how incremental increases in carbohydrate, and decreases in fat, affect plasma SFA and palmitoleic acid in adults with metabolic syndrome who were carefully fed moderately hypocaloric diets for 21 wk. A primary hypothesis was that, despite consuming substantially higher amounts of saturated fat, plasma SFA would remain unchanged in the context of lower carbohydrate intake. Plasma palmitoleic acid was hypothesized to decrease sharply after a very low carbohydrate diet and gradually increase as a function of incremental increases in carbohydrate intake.

## Methods

### Experimental Approach

**Figure 1:**
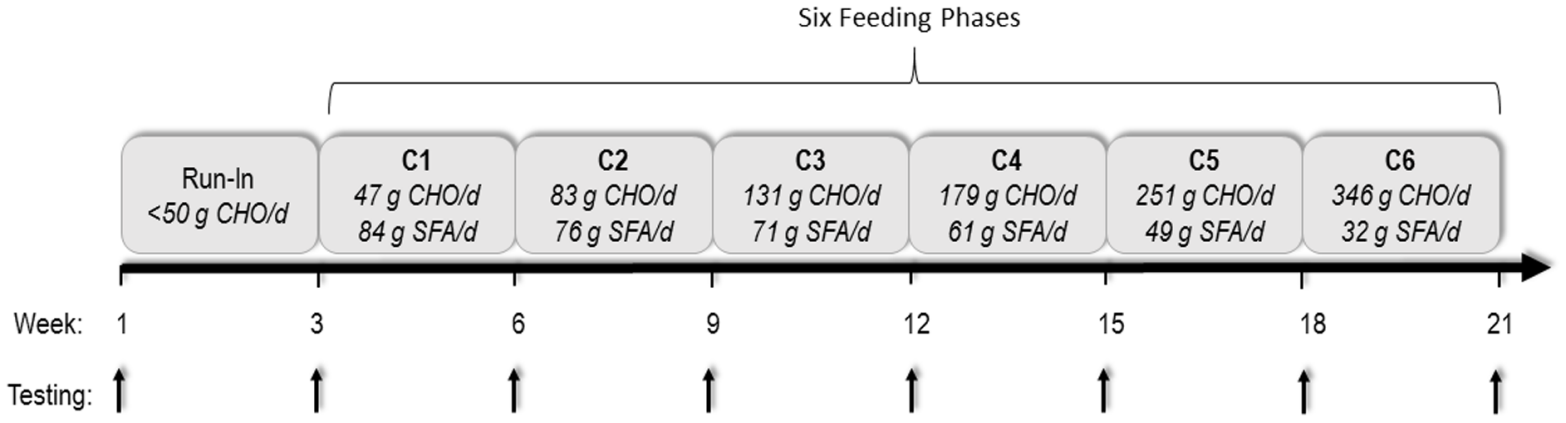
Experimental approach. CHO = carbohydrate, SFA  =  saturated fat.

The experimental design involved a single arm, mildly hypocaloric, ∼18-wk feeding intervention (following a 3-week run-in diet) that incrementally adjusted carbohydrate and fat levels over six 3-wk phases (**Fig 1**). Participants (*n = 16*) were fed a low carbohydrate diet first and gradually transitioned to a high carbohydrate diet over six sequential phases (C1→C2→C3→C4→C5→C6) during which time saturated fat was decreased. Blood was collected at baseline, after the run-in diet, and after each phase (before transition to the next diet) to determine fatty acid composition and other blood-borne markers.

### Study Participants

Sixteen overweight/obese men and women 30-66 years old, with a BMI between 27–50 kg/m^2^ participated in this controlled dietary intervention (**Table 1**). Participants had metabolic syndrome defined as having three or more of the following criteria: waist circumference (≥101.6 cm men, ≥88.9 cm women), blood pressure (≥130/85 mm Hg) or current use of antihypertensive medication, and fasting plasma glucose (≥100 mg/dL), triglycerides (≥150 mg/dL), and HDL-C (<40 mg/dL men, <50 mg/dL women). Subjects were medically screened and excluded if they had a diagnosis of Type I or II Diabetes, liver, kidney, or other metabolic or endocrine dysfunction. Subjects were excluded if they were trying to lose weight or had a body mass change of ±3 kg in the previous 3 mo. Physically active participants were asked to maintain their same levels of activity (verified by activity records) and sedentary individuals did not begin an exercise program in order to limit possible confounding effects on the dependent variables. Subjects were informed of the purpose and possible risks of the investigation prior to signing an informed consent document approved by the University of Connecticut Institutional Review Board who approved this study.

**Table 1:** Baseline subject characteristics^1^.

Sex (M/F)		12/4
Age (yr)		44.9±9.9
Body mass (kg)		108.4±15.1
BMI (kg/m^2^)		37.9±6.3
Waist Circumference (cm)		116.8±10.5
Body Fat (%)^2^		40.0±3.2
Total cholesterol (mg/dL)		191±34
LDL-C (mg/dL)		123±27
HDL-C (mg/dL)		42±8
Triglycerides (mg/dL)		134±54
Glucose (mg/dL)		100±5
Insulin (pmol/L)		93±70
Insulin Resistance (HOMA)^3^		3.3±2.0
Ketones (mmol/L)		0.11±0.06
Systolic Blood Pressure (mmHg)*		122±10
Diastolic Blood Pressure (mmHg)*		83±11
Plasma Triglyceride (wt%)		
	14∶0	1.47±0.44
	16∶0	25.22±2.68
	18∶0	3.69±0.94
	16∶1	3.87±0.90
	18∶1	35.98±2.24
	Total SFA	31.05±3.56
	Total MUFA	40.81±2.44
Plasma Cholesteryl Ester (wt%)		
	14∶0	1.07±0.90
	16∶0	11.03±1.44
	18∶0	1.04±0.61
	16∶1	3.10±1.17
	18∶1	16.00±1.65
	Total SFA	13.69±2.92
	Total MUFA	19.55±2.42
Plasma Phospholipid (wt%)		
	14∶0	0.36±0.14
	16∶0	26.32±1.95
	18∶0	14.76±1.57
	16∶1	0.77±0.22
	18∶1	8.76±0.92
	Total SFA	43.51±1.90
	Total MUFA	11.50±0.98

*^1^Values are mean ± SD (range).*

*^2^Determined by dual-energy X-ray absorptiometry.*

*^3^HOMA  =  homeostatic model assessment  =  [fasting glucose (mmol/L) x insulin (mU/L)]/22.5.*

*5 subjects were using anti-hypertensive medications.

### Dietary Intervention

Six diets were developed that spanned a range of carbohydrate from approximately 50 to 350 g/day (C1→C2→C3→C4→C5→C6) using nutrient analysis software (Nutritionist Pro, Axxya Systems, Stafford, TX). The highest carbohydrate phase (C6) was designed to model national dietary recommendations. While carbohydrate was adjusted every 3 wk, total fat decreased proportionately so that total energy remained constant. Saturated fat was 40% of total fat for all phases. Protein was constant at 1.8 g/kg reference body weight determined from midpoint of Metropolitan Height-Weight Tables. Based on individual resting metabolic needs and activity factors (1.2–1.5) obtained at baseline, the diets were designed to provide a 300 kcal/day energy deficit to induce moderate weight loss and motivation for subjects to continue participation in the 21 wk experiment. Thus, total caloric and protein intake for each individual did not change throughout the study. Estimated nutrient composition of select diets showed high concordance with chemical analysis (Exova, Portland, OR).

For each diet phase 7-day rotational menus were developed that included a wide range of whole foods. Beef, eggs, and dairy were used daily throughout all diet phases as primary sources of saturated fat. For the low carbohydrate diet phases, higher-fat beef and meats, whole eggs, and full-fat dairy products (e.g., cheese, whole milk yogurt, cream, butter) were emphasized. For the higher carbohydrate diet phases with lower saturated fat, leaner versions of beef, egg substitutes, and low-fat dairy (e.g., reduced-fat dairy, skim milk, low-fat/non-fat yogurt) were used instead. Whole grain and relatively low glycemic index carbohydrate sources were emphasized.

Prior to the 18-wk controlled feeding intervention, subjects were counseled to consume a 3-wk very low-carbohydrate run-in diet that mirrored the first low-carbohydrate feeding phase (C1, ∼50 g carbohydrate/day) in order to initiate metabolic adaptations to carbohydrate restriction. Three-day diet records were utilized to determine nutrient intake prior to baseline and during the run-in diet.

Following the run-in period, subjects were provided with all food for 18 wk, which was prepared and packaged by staff in our research kitchen. Food was packaged and labeled per individual serving sizes based on individual caloric and macronutrient needs and was picked up by the subjects 3-4 times/wk. If unable to travel to obtain the food, arrangements were made to ensure the subject received his/her food as planned. No other foods or beverages were allowed except very-low/non-caloric products (e.g., coffee, tea, water, diet soda). All food containers were returned unwashed and inspected to document that all food was consumed.

### Measurements

Subjects arrived to the laboratory following a minimum 12-hr fast and 24-hr abstinence from exercise, caffeine, over the counter medications, and alcohol. Body mass was measured using a digital scale (Ohaus Corp., Parsippany, NJ), height was measured with a stadiometer, and waist circumference was assessed using a standard tape measure performed by the same person. Resting energy expenditure and substrate oxidation was measured by indirect calorimetry (Parvomedics TrueOne 2400 metabolic cart) in a thermal neutral room. The metabolic carts were calibrated with a standard gas mixture each morning. Subjects relaxed quietly for approximately 30 min and oxygen consumption (VO_2_) and carbohydrate expiration (VCO_2_) were averaged for 15 min to determine respiratory exchange ratio (VCO_2_/VO_2_). Prior to blood collection, subjects provided a small urine sample to assess specific gravity as a measure of hydration. Subjects with a USG≥1.025 were asked to consume water and wait 30 min before another urine sample was measured.

Blood samples were obtained from an arm vein after subjects rested quietly for 15 min in the supine position. Whole blood was collected into tubes with a serum separator and ethylenediaminetetraacetic acid (EDTA). Tubes with serum separator remained at room temperature for 15 min prior to centrifugation to allow clotting to occur. Whole blood was centrifuged at 1500×g for 15 min and 4°C, aliquoted into storage tubes, and stored in ultra-low freezers for batch analysis. A portion of serum (∼7 mL) was immediately sent on ice to a certified medical laboratory (Quest Diagnostics, Wallingford, CT) for determination of glucose, total cholesterol (TC), HDL-C, TG, and calculated LDL-C concentrations using automated enzymatic procedures (Olympus America Inc., Melville, NY). Subjects returned to the laboratory for a second fasting blood draw 24–48 hr after visit 1 to repeat glucose and lipid testing. The results from both days were averaged to account for day-to-day variability.

### Blood Analysis

Frozen samples were thawed only once before analysis. Serum insulin was analyzed in duplicate by ELISA (ALPCO, Salem NH). Intra- and inter-assay coefficient of variation (CV) were 5.3 and 7.2% respectively. Glucose and insulin values were used to calculate an index of insulin resistance [HOMA-IR; calculated as Glucose (mmol/L)·Insulin (µIU/mL)/22.5] [26]. Total ketones were determined by a cyclic enzymatic method that measures both acetoacetate (AcAc) and 3-hydroxybutyrate (3-HB) (Wako Chemicals USA Inc, Richmond, VA) with a sensitivity of 1.2 µmol/L, and intra- and inter-assay CV 7.7 and 20.3% respectively. Plasma TG, CE, and PL were extracted and analyzed for fatty acid methyl ester composition using capillary gas chromatography as previously described [24]. Fatty acids were identified by comparison to authentic fatty acid standards and quantitated with peak area and internal standard and expressed as percent weight (wt%).

### Statistical Analyses

One subject dropped after completing C4 due to a rise in his blood pressure. The C5 and C6 data were interpolated based on mean percent changes for the group. A paired samples t-test was used to examine the effects of 6-wk of very low carbohydrate intake (Baseline vs C1). A repeated measures analysis of variance (ANOVA) was used to assess changes across the six diet phases (C1→C2→C3→C4→C5→C6) and Fisher's LSD post hoc was used to examine pairwise comparisons when significant main effects were observed. The alpha level for significance was set at p ≤0.05.

## Results

### Dietary Intake

Total daily energy intake at baseline showed an average consumption of 3028 kcal/day with habitual carbohydrate intake at 44% of total energy, slightly lower than the average American diet, while saturated fat intake was 14% of total energy, slightly higher than the average American diet (**Table 2**). Energy intakes during the controlled feeding periods averaged slightly over 2,500 kcal/day, which was consistent with the goal to create a moderate caloric deficit. As designed, energy and protein intakes across the 6 diet phases were constant for each person. Compared to baseline (46 g/day), saturated fat intake was nearly doubled at C1 (84 g/day) and then gradually decreased to levels below baseline at C6 (32 g/day). From C1 to C6, carbohydrate increased from 47 to 346 g/day corresponding to 7 and 55% of total energy, respectively. All diets were well tolerated and compliance was high based on verbal communication and inspection of returned, unwashed food containers.

**Table 2:** Daily nutrient intakes at baseline (habitual diet) and during each dietary phase^1^.

		Controlled Feeding Phases					
	Baseline^2^	C1	C2	C3	C4	C5	C6
Energy (kcal)	3028±1049	2553±327 2528±339		2585±286	2506±333	2517±339	2509±336
Protein (g)	132±44	129±7	125±6	125±8	123±10	123±10	123±11
Carbohydrate (g)	333±149	47±2	83±4	131±3	179±2	251±12	346±28
Fat (g)	130±44	209±34	193±35	179±29	152±34	121±32	80±27
SFA (g)	46±19	84±14	76±15	71±11	61±15	49±14	32±11
MUFA (g)	33±13	77±14	64±11	57±8	48±11	36±10	24±8
PUFA (g)	20±10	31±5	35±8	35±9	27±7	24±6	14±5
Cholesterol (mg)	533±218	844±96	878±91	824±68	583±131	448±136	334±154
Fiber (g)	27±15	15±1	19±1	23±4	27±3	29±2	35±5

*^1^Values are mean ± SD.*

*^2^Determined from 3-day diet records.*

### Weight and Metabolic Responses

**Figure 2:**
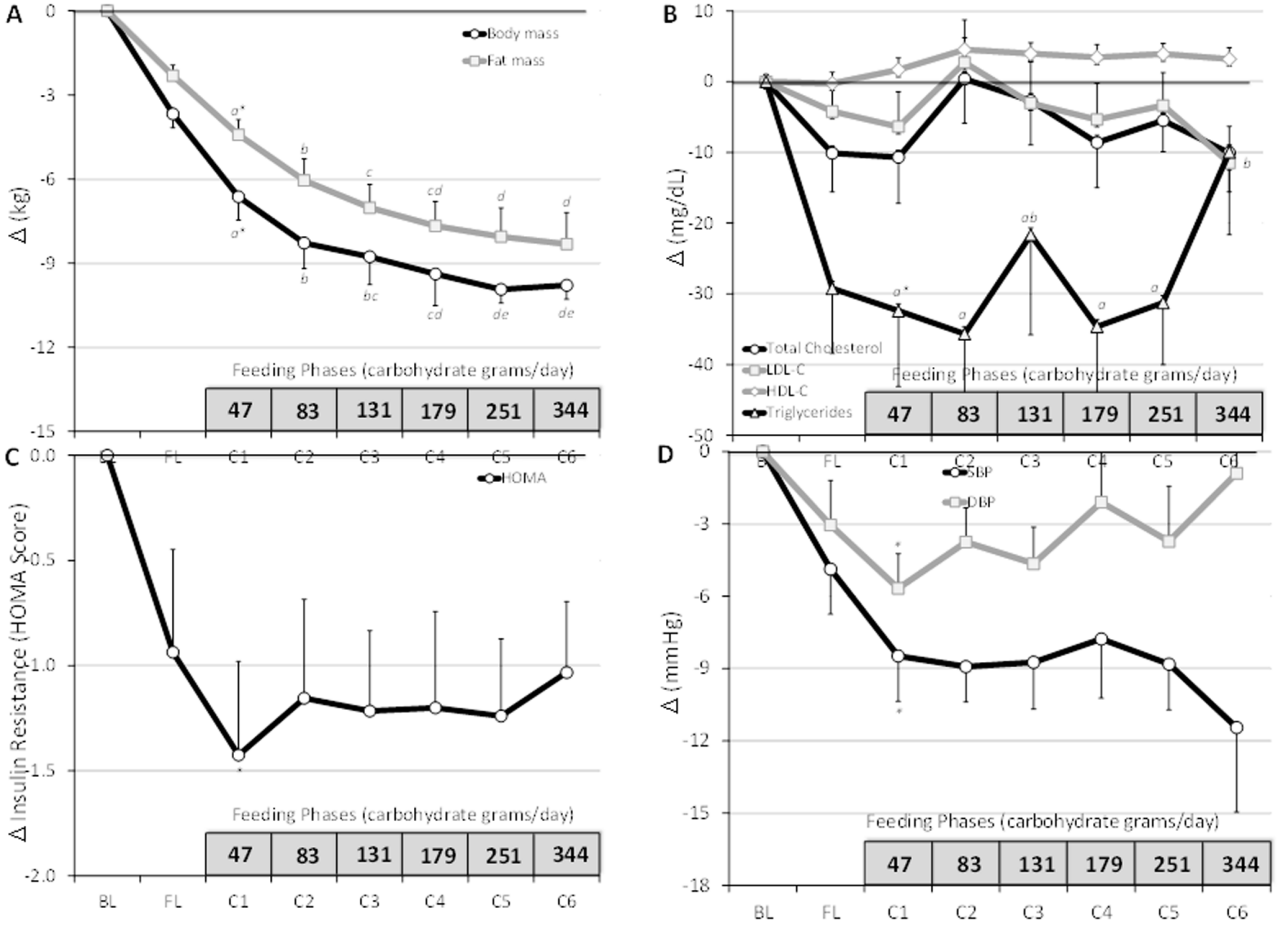
Cumulative change from baseline in (A) body mass and fat mass from dual-energy x-ray absorptiometry, (B) fasting lipoproteins, (C) insulin resistance determined from homeostatic model assessment (HOMA), and (D) blood pressure in 16 subjects who switched to a very low carbohydrate diet and then incrementally increased carbohydrate every 3 wk over six sequential phases (C1→C2→C3→C4→C5→C6). BL  =  baseline, FL  =  free-living low-carbohydrate diet). Significant differences from Baseline vs C1 were determined by dependent t-test and indicated by an asterisk. Differences from C1 to C6 were determined by repeated measures ANOVA and Fisher's LSD post hoc. Different letters at a time point indicate statistical significance.

Relative to baseline, cumulative mean weight loss after the free-living, C1, C2, C3, C4, C5 and C6 phases was -3.7, −6.6, −8.3, −8.8, −9.4, −9.9, and −9.8 kg, respectively, the majority of which was comprised of fat (**Fig 2A**). Serum TG concentrations were lower from C1 to C5 and increased to levels similar to baseline at C6 (**Fig 2B**). Total serum cholesterol, LDL-C and HDL-C were not significantly different across all diets. Compared to baseline, serum glucose (P = 0.048), insulin (P = 0.004), HOMA (P = 0.006) (**Fig 2C**), systolic (P = 0.000) and diastolic (P = 0.001) blood pressure (**Fig 2D**) were significantly lower at C1, but were not significantly different across the 6 diet phases. Compared to baseline, serum ketones increased approximately 5-fold during C1, 3-fold after C2, 2-fold after C4 and returned to baseline levels by C5. Respiratory exchange ratio significantly decreased from baseline to C1 (0.80±0.05 to 0.75±0.04), and then increased linearly at C2, C3, C4, C5, and C6 (0.77±0.03, 0.79±0.04, 0.80±0.04, 0.82±0.03, 0.84±0.05, respectively) with values at C3, C4, C5, and C6 being significantly higher than C1.

### Saturated Fatty Acids

Despite a marked increase in dietary saturated fat intake from baseline to C1 (46 to 84 g/day) and then a progressive decrease to 32 g/day at C6, the proportion of total SFA in plasma TG, CE, and PL were not significantly affected (**Table 3**). From C1 to C6 when dietary saturated fat decreased from 84 to 32 g/day, it is noteworthy that plasma total SFA actually increased in 56%, 88%, and 56% of participants in the TG, CE, and PL fractions. Plasma 16∶0, the predominant saturated fatty acid, was not significantly different over time in the TG and CE fractions. Compared to C1, plasma 16∶0 in the PL lipid fraction was significantly decreased at C2, C3, C4, and C5 but not at C6. Plasma 14∶0 is a minor constituent of total fatty acids but in all three plasma lipid fractions it showed more consistent and significant decreases with carbohydrate restriction, and subsequent increases with progressively higher levels of carbohydrate. As carbohydrate increased, plasma 18∶0 decreased in the TG fraction, but increased in the PL fraction, although the absolute changes were relatively modest.

**Table 3:** Plasma fatty acid responses^1^.

		Controlled Feeding Phases^2^						P-Value	
	Free-Living^3^	C1	C2	C3	C4	C5	C6	T-Test^4^ ANOVA*^5^*	
Triglyceride (wt%)									
14∶0	1.02±0.30	0.99±0.38*^a^*	1.09±0.33*^a^*	1.28±0.44*^ac^*	1.27±0.75*^ac^*	1.62±0.89*^bc^*	1.79±0.98*^b^*	0.002	0.000
16∶0	23.94±1.95	24.36±1.63	23.96±1.15	24.34±2.01	24.92±3.51	25.50±3.63	25.65±4.15	0.268	0.150
18∶0	4.06±0.92	4.00±0.78*^a^*	3.84±0.75*^ac^*	3.82±0.75 *^ac^*	3.73±0.83*^a^*	3.61±0.75*^bc^*	3.45±0.63*^b^*	0.078	0.027
16∶1	2.92±0.61	2.59±0.58*^a^*	2.79±0.77*^ac^*	3.15±0.92*^bc^*	3.09±1.03*^bc^*	3.61±1.22*^d^*	3.89±1.26*^d^*	0.000	0.000
18∶1	37.04±3.70	36.83±2.76*^a^*	37.13±1.89*^a^*	35.87±2.47*^ab^*	36.25±2.97*^ab^*	35.81±3.31*^ab^*	35.09±3.09*^b^*	0.126	0.050
SFA	29.72±2.54	29.82±1.83	29.45±1.24	29.95±2.24	30.43±4.50	31.19±4.69	31.38±5.31	0.242	0.294
MUFA	40.80±3.90	40.27±3.14	40.81±2.50	39.80±2.43	40.09±2.70	40.17±3.31	39.79±2.95	0.438	0.731
Cholesteryl Ester (wt%)									
14∶0	0.69±0.78	0.65±0.42*^a^*	0.67±0.44*^ab^*	0.90±0.57*^bc^*	0.79±0.41*^abc^*	0.80±0.31*^abc^*	1.01±0.47*^c^*	0.005	0.032
16∶0	11.31±1.62	11.17±1.25	11.07±1.25	10.88±1.32	10.66±0.74	10.66±1.36	11.34±1.00	0.579	0.188
18∶0	0.99±0.40	0.92±0.38	1.01±0.53	1.20±0.84	0.94±0.42	0.96±0.51	1.06±0.58	0.168	0.143
16∶1	1.90±0.50	1.72±0.38*^a^*	2.23±1.35*^b^*	2.69±1.45*^bc^*	2.31±1.00*^b^*	2.40±1.17*^bc^*	2.84±1.28*^c^*	0.000	0.001
18∶1	15.92±1.40	15.98±1.37	15.89±1.37	15.53±2.09	15.49±1.53	15.20±1.85	16.17±1.26	0.963	0.338
SFA	13.39±2.30	13.21±2.12	13.24±2.14	13.62±3.33	12.89±1.68	12.90±2.18	14.34±2.39	0.231	0.113
MUFA	18.23±1.56	17.95±1.42	18.50±2.33	18.73±3.51	18.18±1.97	18.03±2.56	19.44±1.99	0.034	0.258
Phospholipid (wt%)									
14∶0	0.27±0.13	0.30±0.14*^a^*	0.28±0.09*^a^*	0.33±0.10*^ab^*	0.34±0.12*^bc^*	0.38±0.14*^cd^*	0.37±0.11*^bd^*	0.044	0.000
16∶0	26.90±1.63	27.52±1.49*^a^*	26.82±1.72*^b^*	26.29±1.67*^b^*	26.72±1.87*^bc^*	26.24±1.75*^b^*	26.97±2.09*^ac^*	0.001	0.001
18∶0	13.68±1.47	13.13±1.20*^a^*	13.44±1.55*^ab^*	13.79±1.71*^b^*	13.71±1.44*^b^*	13.68±1.59*^b^*	13.88±1.77*^b^*	0.000	0.048
16∶1	0.57±0.17	0.61±0.31*^a^*	0.55±0.16*^a^*	0.60±0.22*^a^*	0.60±0.23*^a^*	0.63±0.20*^ab^*	0.74±0.26*^b^*	0.055	0.030
18∶1	8.09±0.81	8.23±0.59*^a^*	8.05±0.47*^ac^*	8.22±0.82*^a^*	8.37±0.62*^a^*	8.53±0.81*^abc^*	8.75±0.80*^b^*	0.074	0.005
SFA	43.17±2.49	43.22±1.41	42.98±2.17	42.80±1.65	43.10±1.70	43.56±1.65	43.56±1.74	0.489	0.114
MUFA	11.07±1.19	11.21±1.27	11.20±1.52	10.99±1.21	11.05±0.93	11.22±0.90	11.51±1.24	0.421	0.396

*^1^Values are mean ± SD from 16. SFA  =  total saturated fatty acids; MUFA  =  total monounsaturated fatty acids.*

*^2^C1  =  lowest carbohydrate diet and C6  =  highest carbohydrate intake.*

*^3^3wk run-in diet phase before entering feeding portion of study.*

*^4^Dependent t-test (Baseline vs C1).*

### Monounsaturated Fatty Acids

**Figure 3:**
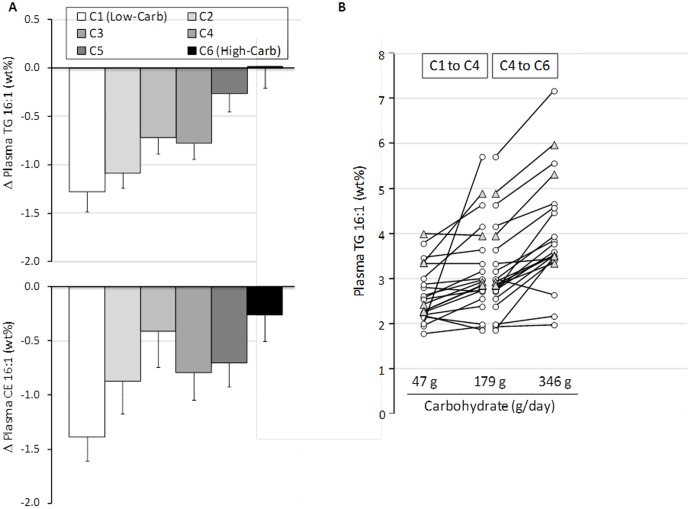
(**A**) Change from baseline in plasma palmitoleic acid (16∶1) in triglyceride (TG) and cholesteryl ester (CE) in subjects who consumed a very low carbohydrate diet (C1) and then gradually transitioned to a high carbohydrate diet over six sequential phases (C1→C2→C3→C4→C5→C6). (**B**) Individual responses in plasma TG 16∶1 from C1 to C4 to C6 corresponding to 47, 179, and 344 g carbohydrate/day. Open circles are subjects who went from low- to high-carbohydrate, and shaded triangles are subjects who went from high- to low-carbohydrate intake.

Compared to baseline, palmitoleic acid was significantly (P = 0.000) decreased at C1 in plasma TG (−33%) and CE (−49%) fractions and the response was highly uniform, with all subjects showing a decrease from baseline to C1. Plasma TG and CE palmitoleic acid showed a step-wise increase as carbohydrate was progressively increased from C1 to C6 (**Fig 3A**). The subjects demonstrated a wide range of palmitoleic acid levels at any given carbohydrate intake; however there was reduced variance with lower carbohydrate diets (**Fig 3B**). There was also a noticeable uniformity among subjects in their progressively higher palmitoleic acid levels going from low- to moderate- to high-carbohydrate intakes. Similar to 18∶0, as carbohydrate increased plasma oleic acid (18:1n-9) decreased in the TG fraction, but increased in the PL fraction, although the absolute changes were small.

## Discussion

A cornerstone of dietary guidelines has been the restriction of saturated fat, but that position is now being questioned in large part because recent analyses have found that saturated fat intake is unrelated to risk of disease [2]. However, a higher proportion of plasma saturated fat is related to increased risk of diabetes and heart disease. Thus, there is a need to better understand the relationship between dietary and plasma saturated fat. In this study, we sought to shed light on the impact of replacing saturated fat with carbohydrate on plasma fatty acid composition. Subjects were studied over 21 wk while consuming diets that were progressively higher in carbohydrate and lower in fat. This ‘dose response’ protocol involved six levels of carbohydrate, which allowed us to examine how a wide range of carbohydrate increments affected plasma fatty acid composition within the same person. The results showed that increasing intake of dietary saturated fat did not accumulate in plasma lipid fractions when carbohydrate was restricted, and moreover when dietary saturated fat intake was decreased there was not a consistent decrease in plasma saturated fat. Whereas plasma saturated fat did not associate with dietary carbohydrate or saturated fat; plasma palmitoleic acid, a biomarker associated with increased risk of hyperglycemia, insulin resistance, metabolic syndrome, and type-2 diabetes, tracked incrementally with dietary carbohydrate.

Several lines of evidence point to endogenously produced palmitoleic acid (i.e., cis-16:1n-7) as being associated with dietary carbohydrate intake. In a large population consuming high-carbohydrate diets, there were incremental increases in erythrocyte palmitoleic acid across quartiles of carbohydrate intake ranging from 273 to 419 g/day [9]. In our previous hypocaloric and isocaloric very low-carbohydrate diet studies, we observed consistent decreases in plasma palmitoleic acid independent of fat composition and weight loss. The current results provide additional data that dietary carbohydrate is a primary driver of plasma palmitoleic acid. Subjects who progressively increased carbohydrate from 47 to 346 g/day showed a step-wise increase in plasma palmitoleic acid.

There was remarkable uniformity in the pattern of plasma palmitoleic acid responses as a function of dietary carbohydrate, although the individual trajectories varied. There was also significant variability between individuals during each diet phase with greater variance as carbohydrate increased (**Fig 3B**). For example, at a carbohydrate intake of 47 g/day TG palmitoleic acid varied from ∼2 to 4 wt%, whereas at 346 g/day it varied from 2 to 7 wt%. Higher proportions of palmitoleic acid in blood or adipose tissue are consistently associated with a myriad of undesirable outcomes such as obesity [27], [28], hypertriglyceridemia [29], hyperglycemia [15], inflammation [30], [31], metabolic syndrome [8], [9], [30], type-2 diabetes [10]–[14], coronary disease [32], heart failure [33], and incidence and aggressiveness of prostate cancer [21]. It is difficult to assign a specific threshold above which palmitoleic acid confers an increased risk of developing these conditions. In the Physician's Health Study a one standard deviation increase in plasma palmitoleic acid was associated with a 17% higher odds ratio of congestive heart failure [33]. In obese subjects who lost significant weight, higher adipose tissue palmitoleic acid before the diet was associated with failure to maintain weight loss [27]. Since the presence of palmitoleic acid is an indicator of *de novo* fatty acid synthesis [3], as there is little palmitoleate in common dietary fats, its rising proportion may serve as a proxy of carbohydrate flux through non-oxidative disposal pathways and a harbinger of adverse clinical outcomes.

In regards to total plasma SFA, the pattern of response was more variable than palmitoleic acid. Similar to our previous studies [24], [25], when dietary saturated fat was increased in the context of a very low-carbohydrate intake, the proportion of total plasma SFA was not increased. In this study saturated fat intake at baseline was increased by 38 g/day at C1 through regular consumption of whole eggs, full fat dairy, and high-fat meats. The lack of accumulation of this additional saturated fat was likely due in part to greater oxidation of SFA, as indicated by the significant decrease in respiratory exchange ratio during C1. Whole body fat oxidation increases markedly when dietary carbohydrate is restricted [34], and it is likely that SFA become preferred substrates for beta-oxidation in low-carbohydrate-adapted individuals.

Carbohydrate-induced insulin secretion stimulates DNL and potently suppresses lipolysis and fat oxidation [35], which would promote accumulation of endogenous SFA even when they are reduced in the diet. In the current study, dietary saturated fat was decreased by more than half from C1 to C6 (84 to 32 g/day), yet the majority of participants showed a numerical increase in plasma SFA in all lipid fractions over this same time period. The relative contribution of DNL and fat oxidation and their sensitivity to dietary carbohydrate manipulation likely varies considerably between people and explains the less uniform response in total plasma SFA observed in the current study. However, the pattern of lower plasma SFA after the low-carbohydrate diet with the highest amount of saturated fat, and numerically higher plasma SFA after the high-carbohydrate diet with the least amount of saturated fat, is consistent with the regulation of DNL and fat oxidation by carbohydrate intake and its effect on the glucose-insulin axis.

The reduced proportion of plasma palmitoleic acid after the low-carbohydrate diet was associated with positive responses in other traditional risk markers. Serum triglycerides, glucose, insulin, and estimates of insulin sensitivity were improved as well. Serum cholesterol responses were variable but consistent with the known effects of carbohydrate restriction to increase, on average, total cholesterol, HDL-C and LDL-C relative to low-fat diets [36].

There were several limitations in this study. The diet phases were relatively short to keep the entire feeding portion of study less than 6 months, and by design we created menus that were hypocaloric to induce weight loss. The highest carbohydrate intake was only 55% of total energy and it was consumed in the context of a daily caloric deficit and ongoing weight loss for most subject. Whether carbohydrate-induced increases in plasma palmitoleic acid would have been similar or more pronounced in the context of eucaloric weight maintenance diets remains unknown. Furthermore, since subjects initially restricted carbohydrates and then sequentially added them back over time it is difficult to disassociate temporal changes that may be influenced by cumulative weight loss or lingering effects from the previous diet phases. To address this limitation we provided the diets in reverse order (i.e., high- to low-carbohydrate) to a small number of participants (n = 5). In these individuals, plasma palmitoleic acid responded in the exact opposite pattern as the primary group providing strong evidence that the major driver of circulating palmitoleic acid was the level of carbohydrate in the diet, and was not significantly modified by the order of diets, length of each diet phase, or weight loss (data not shown).

In summary, high intakes of saturated fat (including regular consumption of whole eggs, full-fat dairy, high-fat beef and other meats) does not contribute to accumulation of plasma SFA in the context of a low carbohydrate intake. A progressive decrease in saturated fat and commensurate increase in carbohydrate intake, on the other hand, is associated with incremental increases in the proportion of plasma palmitoleic acid, which may be signaling impaired metabolism of carbohydrate, even under conditions of negative energy balance and significant weight loss. These findings contradict the perspective that dietary saturated fat per se is harmful, and underscore the importance of considering the level of dietary carbohydrate that accompanies saturated fat consumption.
